# Novel structure in sciaenid fish skulls indicates continuous production of the cephalic neuromast cupula

**DOI:** 10.1038/srep37523

**Published:** 2016-11-23

**Authors:** Maíra Pombo, Alexander Turra

**Affiliations:** 1 University of São Paulo, Oceanographic Institute, São Paulo, São Paulo, Brazil

## Abstract

The presence of a conspicuous and frequent but never-described structure in the skull cavities of sciaenid fish was noted during population studies in an urbanized bay. The ultrastructure closely resembles the cupula of neuromasts, an organ associated with the perception of the environment in teleost fish. The bodies were recorded detached in both preserved and freshly sampled individuals and without associated cilia. Prominent characteristics are acellularity, the elliptic-conic shape composed of stack-like protein lamellas, and a mesh-like appearance in cross section. These acellular lamellar cephalic bodies (ALCBs) were more abundant in larger individuals and showed temporal peaks of abundance independently of the fish size. The conic and lamellar features suggest that the deposition of protein layers follows fish growth, and the bimodality of the size of these structures in individuals indicates temporal peaks of production. These results indicate that these ALCBs are a consequence of the accretion of the cupula of neuromasts at a faster rate than they degrade. Given the novelty of this structure and the increasing records of diseases of marine organisms worldwide, an important question is whether these bodies occur subsequently to some environmental change and whether their accumulation in the skull cavities has consequences to fish health.

This contribution is a report and description of a never-reported but quite conspicuous structure associated with the skull cavities of sciaenid fish. Because this is the first report of the occurrence of these structures, we refer to them, for the moment, as acellular lamellar cephalic bodies (ALCBs), based on their location and most prominent features. During a series of studies with populations of sciaenids in shallow areas of a bay in southeastern Brazil, some small, elliptic-conic stack-like structures were observed loose in the cavities of the cavernous cranial bones that are typical of the family, in all of the more than 3000 individuals examined. An extensive inquiry including literature searches and consultations with specialists did not yield any conclusive information about the nature of these structures. The best guess of specialists consulted was often about parasitism or parasite-related structures, although this possibility was soon discarded for several reasons. The structure that draws most attention in this body region, the cephalic neuromast, is similar in both size and shape to the ALCB. The hypothesis that the two are related came to be regarded as the most probable, although it seems rather unlikely given the available knowledge so far.

The neuromasts, individual organs that constitute the mechanoreceptive system, the lateral line, of all fishes have a pattern of distribution that is species-specific^1^,^2^. In members of the family Sciaenidae, the cephalic lateral line is hypertrophied and located in enlarged canals, both important distinguishing characteristics of this family^3^. Still, not much is known about the specific details of this hypertrophy. In general, these organs develop during the larval stages and lose the ability to regenerate during ontogenesis^4^,^5^. The cupula can grow continuously, to replace the wear of the terminal portion^6^,^7^,^8^; however, the detached structures that we report here vary in number according to fish size and also temporally. For these reasons, a relationship of these ALCBs with neuromasts is debatable.

Soft tissues are usually disregarded in morphological studies of fish, but the conspicuity and location of the ALCBs, their presumable association with neuromasts, and the fact that they have not been described so far, raise the possibility that they result from some kind of disorder.

Here, we report the observation of these acellular lamellar cephalic bodies in a species of Sciaenidae, documenting their structure and the evidence for the hypothesis that they are actually detached cupulas from cephalic neuromasts.

## Methodology

The individuals where the ALCBs were first noticed were collected in southeastern Brazil for a series of ecological studies, which provide detailed information about sampling procedures^9^,^10^,^11^. Briefly, sampling was performed from August 2003 through October 2004 in Caraguatatuba Bay (from 23°43′25.3″S 045°24′07.1″W to 23°37′41.1″S 045°24′02.4″W), in São Paulo state. Two areas were selected, avoiding the influence of continental waters, in which three otter trawls (2 cm mesh size) were deployed monthly from 800 m to 1600 m off the beach, which corresponded to depths of approximately 1 to 4 m. For the purposes of the dietary studies, the fish were immediately preserved in 10% formalin and, after sorting, all the specimens were labeled and fixed in 70% ethanol. All sampling procedures were in accordance with federal law, and were approved by the appropriate federal environmental agency (*Ministério do Meio Ambiente (MMA)*–*Instituto Brasileiro do Meio Ambiente e dos Recursos Naturais Renováveis (IBAMA)-Diretoria de Recursos Naturais Renováveis (DIREN)*, acronyms for, in English: Environment Ministry–Brazilian Institute of Environment and Renewable Natural Resources-Directorate of Renewable Natural Resources), under license No. 31629-1.

During population studies of the most abundant sciaenid, *Stellifer rastrifer*, the structures were noted beneath the skin of the skull. Skin removal revealed that these were loose in the prominent skull cavities that are typical of the family^3^. Therefore, all the specimens were assessed for their occurrence. Of the more than 3000 individuals of the species examined, all showed some number of ALCBs. Other species of Sciaenidae from the area (*Larimus breviceps*, *Paralonchurus brasiliensis*, *Stellifer brasiliensis* and *S. stellifer*) were preliminarily assessed, and all also contained the structure. Since *S. rastrifer* was the most abundant, the species was chosen for subsampling for more detailed analysis.

### Study area

Caraguatatuba Bay is located in the municipality of the same name, which is within an urban center and is also a popular tourist destination. For many decades, the bay has been used for sewage disposal, in amounts that vary widely with the number of seasonal visitors^12^,^13^. Artisanal fishing is still an important economic activity, and at the time of the sampling, about 800 fishing boats were active locally. The municipality is also impacted by oil spills in the São Sebastião Channel, an important site of oil and natural gas exploitation.

The sampling areas in the bay were shallow, at a distance of 800 to 1600 m from the shore at mean low water, which corresponds to depths from 1 to 4 m. The sites were located as far as possible from the influence of the several small rivers entering the bay.

### General Information

The necessary number of ALCBs were removed from the fish skulls and subjected to a variety of investigative techniques. For the cases that required fresh structures for glutaraldehyde fixation (for examination with Transmission Electron Microscopy), the study area was revisited and bycatch specimens were obtained from fishermen.

### Morphological Procedures

Several different microscopy techniques were used to investigate the structure and ultrastructure of the ALCB, in all their dimensions: (i) Stereo Optical Microscope was used for three-dimensional observation of large numbers of ALCBs; (ii) Scanning Electron Microscope (SEM) was used to analyze the surface of the ALCB, externally and internally; and (iii) Transmission Electron Microscope (TEM) was used to obtain the highest magnification of the ALCB.

### Histochemical Procedures

Histochemical procedures followed by light-microscope observation were used to assess the chemical composition and inner structure of the ALCB. Serial sections were stained with (i) Hematoxylin and eosin (H&E), to distinguish acid/negatively charged (e.g. DNA), from basic/positively charged substances (e.g. proteins); (ii) Periodic acid-Schiff (PAS), to detect storage or structural polysaccharides, and glycoconjugates, which are generally associated with cell and organelle membranes; (iii) Von Kossa, used to quantify mineralization, for a preliminary assessment of a possible calcification process; (iv) Picrosirius, for the presence of collagen, able to identify collagen type if combined with polarized light.

### Protein identification

The first assessments indicated the need to further assess the composition of the ALCB. A total protein extract was submitted to SDS-PAGE (denaturing electrophoresis) fractionation, stained with methylene blue, followed by MALDI TOF/TOF (mass spectrometry) analysis. The fish genome was not sequenced at the time, and therefore a peptide mass fingerprinting of selected protein spots was carried out, i.e., the peptides were identified from previously sequenced proteins, available in public databases.

### Quantification and measurement

Assessment of whether, and how, the ALCB abundance and size varied with individuals and time was essential to answer an important question. If the ALCBs are continuously produced and accumulate in the individual’s skull, larger individuals would show higher abundances of these structures, while alterations in the abundance over time could reflect an environmental influence. Correspondingly, temporal differences in the number of ALCBs among different-sized individuals would reflect how these structures behave over time within different population strata.

Specimens of *S. rastrifer* measuring about 4 to 16 cm long were separated into three size categories, small (5 to 8 cm), medium (>8 to 11 cm) and large (>11 to 14 cm). The relationship between the abundance of ALCBs and the size of individuals was assessed. From each category, 20 individuals were randomly selected in each sampling month. Whenever the samples contained individuals of extreme sizes (i.e., smaller than 4 cm or larger than 14 cm), these were also included in the analysis. For each of the 513 individuals selected, the total length (cm) was recorded, the skin was carefully removed from the skull, and all ALCBs were counted. For each size category the annual pattern of distribution was assessed.

Another related question involved the size distribution of the ALCBs in each individual fish. If the size of these bodies corresponds to that of the neuromasts, which grow along with the fish, then the ALCBs would increase in size in larger individuals. Furthermore, this analysis could help to determine if they accumulate over time, i.e., if the degradation rate of ALCBs is lower than the generation rate, and if there are temporal peaks of production.

For this purpose, two months, April and September 2004, considered of key importance due to the local pattern of population dynamics^14^, were chosen for ALCB size assessments. For these periods, the same individuals assessed for ALCB abundance had their ALCBs measured, i.e., 60 individuals for each month, homogeneously distributed across a length range.

## Results and Discussion

**Figure 1:**
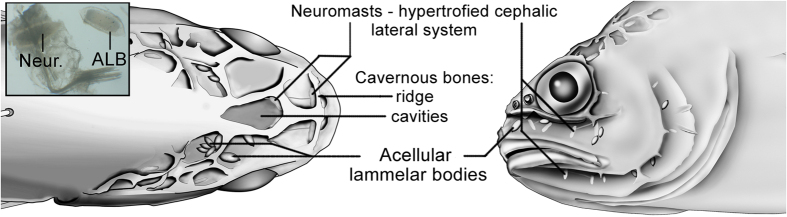
Location, size and shape of ALCBs- resemblance to neuromasts. The scheme shows dorsal and lateral views of the skull of *Stellifer rastrifer* after skin removal. The notable structures are represented: neuromasts and the Acellular Lamellar Cephalic Bodies (ALCB). The photograph highlights the similarity between the shapes of the two structures.

The 100% frequency of occurrence was the first piece of evidence against the hypothesis of parasitism. Also, we found no record in the literature of any similar parasite, although at first glance an ALCB could resemble a worm egg. Furthermore, the most obvious morphological features show that the location (skull cavities), size (about 0.5 to 1 mm in length) and shape (elliptic-conic) of the sciaenid ALCBs resemble those of the cephalic neuromasts (Fig. 1). However, the ALCBs are stack-like structures, released in the cavernous cranium of the fish, and frequently form aggregates.

**Figure 2:**
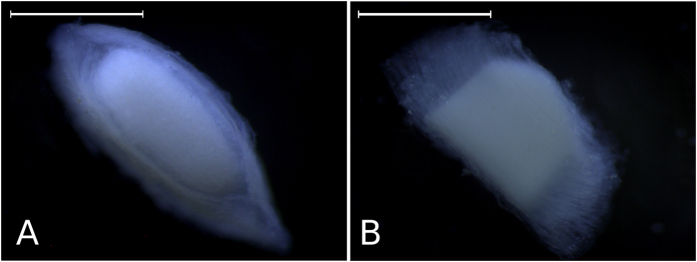
Upper and lateral surfaces. Basic views of the Acellular Lamellar Cephalic Body: upper (**A**) and lateral (**B**) surfaces, under the stereomicroscope. Scales: 1 mm.

**Figure 3:**
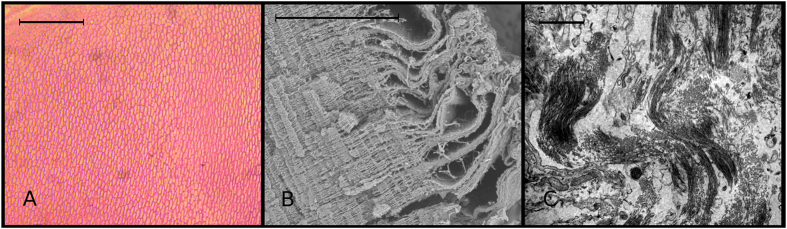
Acellularity. Light (**A**, HE coloration; longitudinal section; scale: 50 μm), scanning (**B**, cross section; scale: 50 μm) and electron (**C**, scale: 0.3 μm) photomicrographs showing the basic ultrastructure of the Acellular Lamellar Cephalic Bodies and the lack of cellular structure.

In the interest of readability, we distinguished three basic surfaces: the lower or basal view, which is the larger surface; the upper surface, very similar at first glance to the basal one, but smaller; and the lateral views, both identical. Both the lower and upper surfaces as viewed under the stereomicroscope are elliptic-conical, with a denser central region surrounded by a more translucent, flap-like area (Fig. 2A). In lateral view, an ALCB is somewhat trapezoidal and shows flap-like sides, with a more opaque central region (Fig. 2B). When describing the ultrastructure of the neuromast cupula, Kelly and van Netten^15^ pointed out that “*the cupula is composed of a central region overlying the macula and wings not overlying the macula”*. These authors also showed that the central region is composed of a series of compartments that lend it “*the appearance of a honey-comb in section*”. Both light and SEM microscopy revealed precisely this feature in the ALCBs assessed here (Fig. 3), an extraordinary resemblance to that shown for the cupula, a structure that, as Kelly and van Netten^15^ mentioned, has a refined structure and is far from being a simple gelatinous mass, as frequently described.

**Figure 4:**
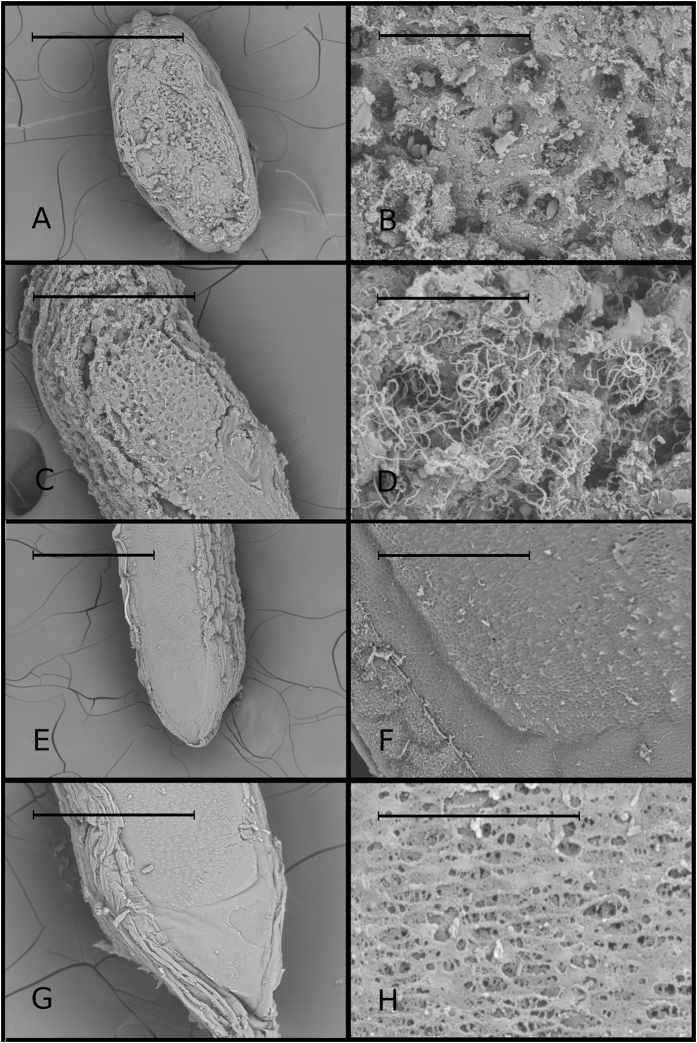
Ultrastructure of upper and lower surfaces. Ultrastructure of upper and lower surfaces of the Acellular Lamellar Cephalic Bodies (scanning electron microscopy). Each row of figures represents a different structure. The upper rows show details of the upper surface of two different ALCBs (scales: (**A**) 500 μm; (**B**) 50 μm; (**C**) 200 μm; (**D**) 30 μm); lower rows (scales: (**E**) 500 μm; (**F**) 50 μm; (**G**) 200 μm; (**H**) 20 μm) show details of the lower surface of two different ALCBs.

In the ultrastructure analysis, the SEM revealed that the upper surface of the structure was in an advanced process of decomposition, also denoted by the presence of abundant bacteria; while the lower view showed a flat, smooth surface (Fig. 4), which indicates that it is more recent. Considering that this undamaged surface is also always the larger, we suggest that the lower surface was most recently in contact with the fish body, i.e., a neuromast surface. The results presented so far suggest that the ALCB is a detached neuromast cupula, which was sequentially produced from the top (older surface) to the bottom (newer surface). If so, this may also explain an important feature, described for the cupula structure, that was not observed for the ALCB: the presence of spherical structures that correspond to associated cilia of the neuromasts. We suggest that the cilia would be associated only with a functional cupula, and the ALCB would represent a non-functional cupula derivative; as new layers are constructed to enclose the cilia, the previous base loses the association with it.

The TEM confirmed that the structure is essentially acellular, and the few cell-like structures observed resemble apoptotic cells. Mainly, the TEM revealed that the ALCB is essentially composed of long, interlaced fibrils (Fig. 3). This procedure required fresh structures for fixation, and in the fresh specimens obtained from fishermen the ALCBs appeared the same as in the fixed specimens, with the exception that in the former they were more translucent. This alone is an important fact because it disproves the hypothesis of detachment of the cupula due to specimen fixation for an extended period of time.

Histochemical procedures did not provide conclusive answers concerning the ALCB composition, but showed their homogeneous nature. No staining technique produced any strong coloration, but only uniform, light stain. Observation of the slides confirmed the acellular nature and mesh-like inner structure of the ALCBs (Fig. 3).

About seven protein bands were isolated by the SDS-PAGE fractionation, from 48.488 to 174.242 KDa. Mass-spectrometry analysis did not identify statistically any sequenced protein, and the sequencing returned no match with global databases, which means that there was no significant correspondence with any protein or gene so far described. An essential further step is to identify this substance through more elaborate chemical techniques. The analysis did show that all fragments had the same mass-to-charge ratio (Supplementary Document S1), indicating that they all belonged to a single protein type. This means that besides being acellular, the ALCB is composed essentially of a single protein.

**Figure 5:**
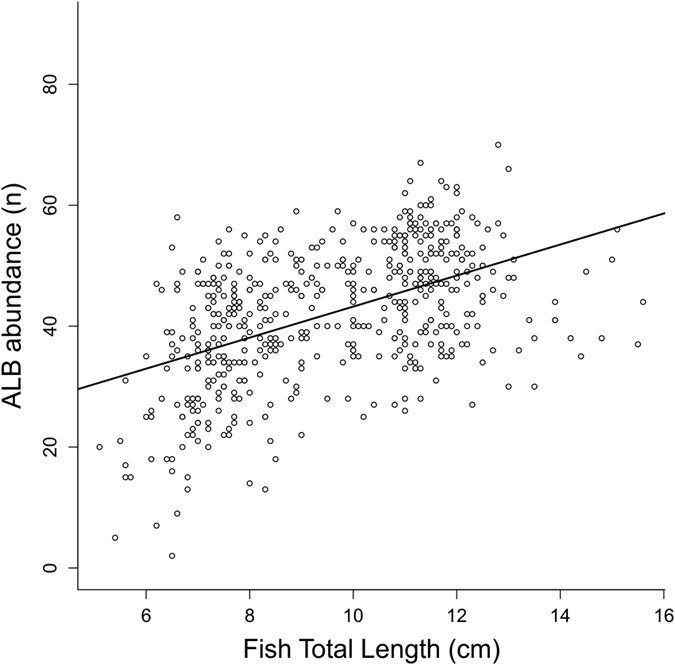
ALCB abundance *versus* individual length. Relationship between the abundance of Acellular Lamellar Cephalic Bodies and the total length (cm) of *Stellifer rastrifer* individuals, sampled from August 2003 through October 2004 in Caraguatatuba Bay, southeastern Brazil.

**Figure 6:**
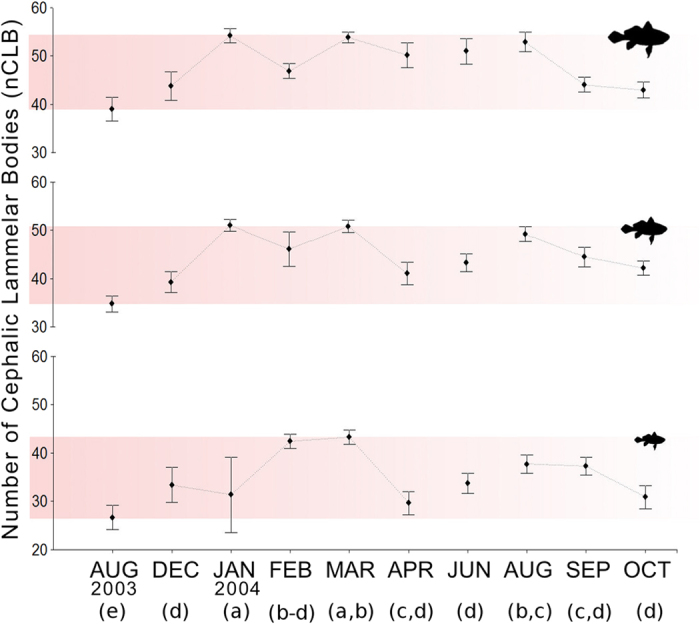
ALCB abundance over time. Temporal distribution of the abundance of Acellular Lamellar Cephalic Bodies for different fish sizes–variable number. Mean and standard error of the number of ALCBs. For each of the ten months (August 2003 through October 2004), 20 individuals of each size class (bottom to top: small–5 to 8 cm, medium – >8 to 11 cm, and large – >11 to 14 cm) of *Stellifer rastrifer*, sampled in Caraguatatuba Bay, were examined. Letters in brackets denote significant differences among months, discriminated by the Tukey test.

Quantification analysis showed a significant positive relationship between the number of ALCBs and the total length of individual fish (Fig. 5), and that, despite fish size, the fish caught in some months had significantly higher amounts (Fig. 6), as denoted by the significant relationship of abundance with both size and date (F_10,500_ = 34.88, p < 0.01, Adj.r^2^ = 0.40), and the lack of a significant interaction of the two (AICs = 3665 and 3652). The size relationship indicates that ALCBs are produced continuously over time, in contrast to the number of neuromasts, which is species-specific and does not increase with increased fish size. In line with the suggested hypothesis, this indicates that the fish does not decompose/release ALCBs at the same rate at which they are produced. The month-to-month variation sheds some light on an important question regarding ALCBs. They may have simply been overlooked so far, or, their occurrence or abundance may have some environmental significance. An important further step is to compare their occurrence and abundance in fish in different areas. The temporal variation does indicate that the production rate of ALCBs is influenced by the environment.

**Figure 7:**
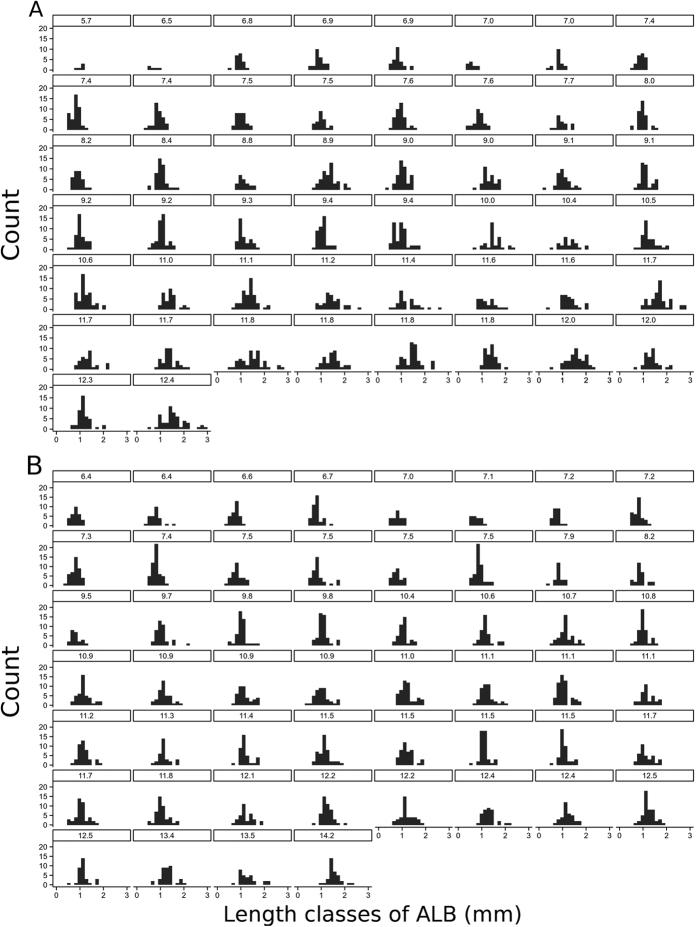
ALCB size distribution. Size distribution of the Acellular Lamellar Cephalic Bodies for different individuals of *Stellifer rastrifer,* collected in Caraguatatuba Bay in (**A**) April and (**B**) September 2004. Each histogram denotes a fish individual, and its title the fish size. Gradual increase in ALCB size with increasing size of fish and frequent emergence of more than one mode support the occurrence of peaks of ALCB production during the life of the fish.

Concerning the size of the ALCBs in each individual fish, the ALCB size distribution tended to become at least bimodal with increased fish size. This feature is shown for two different months: April 2004, when more juveniles entered the study area, and September 2004, when according to the population studies, most young fish had already spent some time growing in the region^11^ (Fig. 7). This result may reflect the history of production and elimination of the ALCBs over the course of the individuals’ life. Each mode is likely to characterize a peak period of production, and the presence of multiple modes further indicates that ALCBs are produced faster than they degrade.

The ontogenesis of the lateral line system has not been broadly investigated, and even less is known of the cephalic lateral line of Sciaenidae specifically. Thus, the present observations raise several questions. For instance, it has been shown that neuromasts may grow slightly after the fish hatch^16^, so, how long do these hypertrophied cephalic neuromasts grow along with the fish? If they grow considerably, it is reasonable to assume that the cupula must be replaced. But at what rate? To answer this question, the mechanisms involved in the production and detachment of neuromast cupulas, and if their presence, especially the sequence and abundance seen here is a regular occurrence must be further assessed. If not, some factor that remains to be identified is overstimulating their production.

Also, if such an occurrence is not normal, what are the reasons for and the implication of the agglomeration of detached cupulas in the skull cavities of the fish? It is important to consider the possible consequences to the fish health, since this accumulation occurs in a compartment with a sensory role.

The sampling area comprised shallow and non-estuarine waters, subjected to several environmental impacts, and new evidence of damage to the natural environment emerges continually. One of the greatest challenges of this century is recognizing and understanding these increasingly frequent alterations^17^,^18^. In the light of these results, we suggest that the so-called ALCB are actually cupulas of cephalic neuromasts, and hypothesize that these individuals underwent some kind of hypersensitization that led to the cupula overproduction. To verify this hypothesis it is important to understand what mechanisms are being activated in the production of these structures, why they form, and what are the possible consequences for the fish’s health.

## Additional Information

**How to cite this article**: Pombo, M. and Turra, A. Novel structure in sciaenid fish skulls indicates continuous production of the cephalic neuromast cupula. *Sci. Rep.* **6**, 37523; doi: 10.1038/srep37523 (2016).

**Publisher's note:** Springer Nature remains neutral with regard to jurisdictional claims in published maps and institutional affiliations.

## Supplementary Material

Supplementary Data S1
