# Supplementary material for: Novel structure in sciaenid fish skulls indicates continuous production of the cephalic neuromast cupula
Source: Sci Rep. 2016 Nov 23;6:37523. doi: 10.1038/srep37523 (PMC5120346; doi:10.1038/srep37523)
Supplement: Supplementary Data S1 [file srep37523-s1.pdf]

Supplementary Data S1. Mass spectrometry report. Protein identification by peptide mass fingerprinting.

In: **Novel structure in sciaenid fish skulls indicates continuous production of the cephalic neuromast cupula**

By: Maíra Pombo and Alexander Turra

Date: 28-05-2012

Target:

Protein identification by peptide mass fingerprinting.

Relevant information:

Peptide mass fingerprinting of protein selected spots was carried out by in-gel trypsin treatment (Sequencing-grade Promega) overnight at 37^{0}C. Peptides were extracted from the gels using 60% acetonitrile in 0.2% TFA, concentrated by vacuum drying and desalted using C18 reverse phase micro- columns (OMIX Pippete tips, Varian). Peptide elution from micro-column was performed directly into the mass spectrometer sample plate with 3 μl of matrix solution(α–cyano-4-hydroxycinnamic acid in 60% aqueous acetonitrile containing 0.2% TFA)
Mass spectra of digestion mixtures were acquired in a 4800 MALDI-TOF/TOF instrument (Applied Biosystems) in reflector mode and were externally calibrated using a mixture of peptide standards (Applied Biosystems). Collision-induced dissociation MS/MS experiments of selected peptides were performed.
Proteins were identified by NCBInr database searching with peptide m/z values using the MASCOT program and using the following search parameters: monoisotopic mass tolerance, 0.05 Da; fragment mass tolerance, 0.25 Da; methionine oxidation, as possible modifications and one missed tryptic cleavage allowed.

Summary of results:

Using MS and MS / MS data, the following proteins were identified:

Sample identification	Mol. Mass (kDa.)	Protein identified by MS	Result found in: (page number)
1	12.0121185	“human keratin”	3-4
2	48.48833315	Unidentified	5
3	56.62034992	Unidentified	6
4	73.31587789	Unidentified	7
5	105.2722064	Unidentified	8
6	139.881978	Unidentified	9
7	174.2422343	Unidentified	10

Sample: 1

Reflector positive ion mode MALDI-TOF-MS spectrum

(Select image and zoom in to see it correctly)

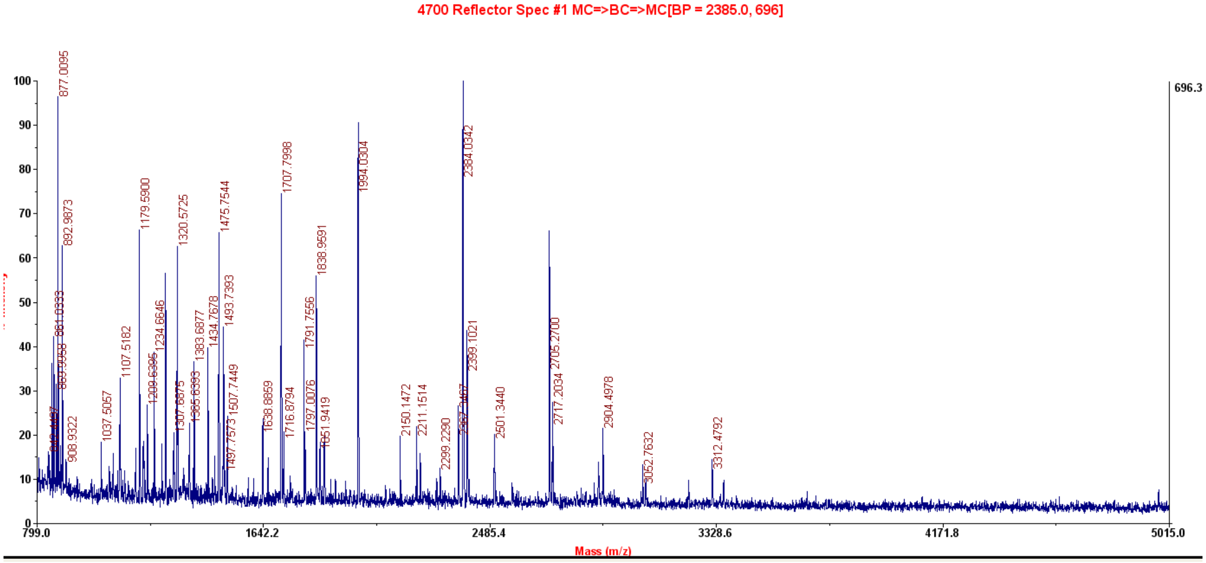

MS (m/z) y MS/MS (m/z) mass list obtained experimentally:

869.9958, 877.0095, 892.9873, 908.9322, 1037.5057, 1082.0354, 1107.5182, 1165.5664
1179.5900 ions(175.1432, 232.1784, 293.1486, 303.2118, 353.2772, 387.3039, 404.2138, 422.2151, 517.4302, 535.3116, 552.3682, 572.2853, 606.3444, 645.4016, 646.5872, 663.3605, 758.5406, 759.6008, 859.5269, 882.8774, 887.5879, 887.8826, 955.0898, 959.9779, 960.2982, 1005.5904)
1193.6056, 1196.6094, 1209.6395, 1234.6646, 1263.6880, 1277.7000, 1307.6875, 1308.6710, 1320.5725, 1323.6310, 1345.6754, 1365.6393, 1379.6691, 1383.6877, 1434.7678, 1460.7717, 1475.7544, 1491.7465, 1493.7393, 1497.7573, 1507.7449, 1638.8859, 1657.8152, 1707.7998, 1716.8794, 1791.7556, 1797.0076, 1837.9988, 1838.9591, 1851.9419, 1867.9514, 1994.0304, 2150.1472, 2211.1514, 2225.1929, 2299.2290, 2367.3467, 2384.0342, 2399.1021, 2501.3440, 2705.2700, 2717.2034, 2889.5222, 2904.4978, 3052.7632, 3312.4792

Search Parameters

```
Type of search          : Sequence Query
Enzyme                  : Trypsin
Variable modifications  : Oxidation (M)
Mass values             : Monoisotopic
Protein Mass            : Unrestricted
Peptide Mass Tolerance  : ± 0.08 Da
Fragment Mass Tolerance: ± 0.3 Da
Max Missed Cleavages    : 1
Instrument type         : MALDI-TOF-TOF
Number of queries       : 55
```

Mascot search engine result (www.matrixscience.com)

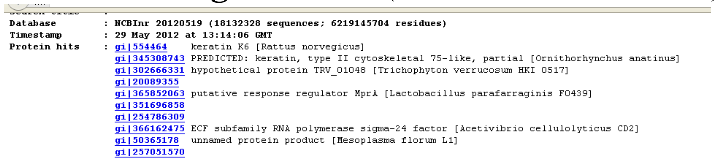

```
Database      : NCBInr 20120519 (18132328 sequences; 6219145704 residues)
Timestamp     : 29 May 2012 at 13:14:06 GMT
Protein hits  : gi|554464    keratin K6 [Rattus norvegicus]
                gi|345308743 PREDICTED: keratin, type II cytoskeletal 75-like, partial [Ornithorhynchus anatinus]
                gi|302666331 hypothetical protein TRV_01048 [Trichophyton verrucosum HKI 0517]
                gi|20089355
                gi|365852063 putative response regulator MprA [Lactobacillus parafarraginis F0439]
                gi|351696858
                gi|254786309
                gi|366162475 ECF subfamily RNA polymerase sigma-24 factor [Acetivibrio cellulolyticus CD2]
                gi|50365178  unnamed protein product [Mesoplasma florum L1]
                gi|257051570
```

Mascot Score Histogram

Ions score is -10*Log(P), where P is the probability that the observed match is a random event.
Individual ions scores > 56 indicate identity or extensive homology (p<0.05).
Protein scores are derived from ions scores as a non-probabilistic basis for ranking protein hits.

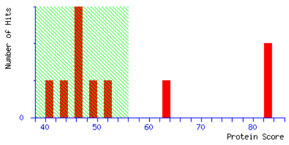

Link to result:
http://www.matrixscience.com/cgi/master_results.pl?file=../data/20120529/FtGooeSEt.dat

Link to protein summary:
http://www.matrixscience.com/cgi/master_results.pl?file=..%2Fdata
%2F20120529%2FFtGooeSEt.dat&REPTYPE=protein&_sigthreshold=0.05&REPORT=10&_m
inpeplen=7&_server_mudpit_switch=99999999&_ignoreionsscorebelow=0&_showsubsets=0&_
showpopups=TRUE&_sortunassigned=scoredown&_requireboldred=0

Link to protein view:
http://www.matrixscience.com/cgi/protein_view.pl?file=..%2Fdata
%2F20120529%2FFtGooeSEt.dat&hit=1

Conclusion:

- Statistically significant identification of the protein "human keratin"

Sample: 2

Reflector positive ion mode MALDI-TOF-MS spectrum

(Select image and zoom in to see it correctly)

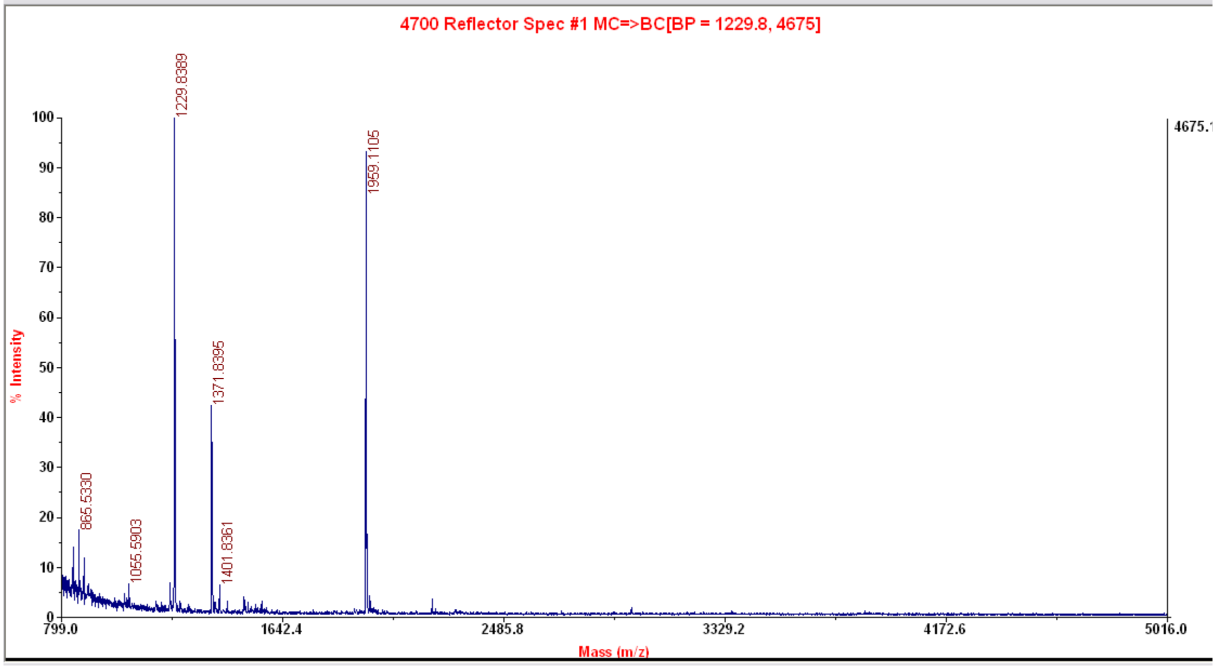

MS (m/z) y MS/MS (m/z) mass list obtained experimentally:

1225.7230
1227.7544
1229.7684 ions(175.1259, 213.0809, 394.3154, 412.3505, 430.3537, 448.3586, 561.4043, 563.3417, 579.4185, 581.3370, 583.2356, 597.4268, 599.3563, 601.1374, 613.4111, 615.4380)
1371.7687 ions(175.1328, 183.1581, 185.1857, 211.1451, 212.1340, 213.1769, 288.2143, 308.2065, 310.2223, 326.2534, 330.1756, 338.1851, 345.2364, 358.1675, 423.3017, 456.1999, 459.2279, 473.2747, 487.2367, 560.3326, 574.2672, 600.3222, 674.3021, 684.3398, 689.3914, 702.3636, 759.3413, 812.4814, 815.4120, 872.4603, 889.4328, 891.4923, 932.5020, 949.5045, 1004.5694, 1011.5371, 1012.5400, 1029.5627, 1046.5961, 1142.6609, 1160.7257)
1959.0148 ions(175.1193, 185.1010, 199.1216, 201.1248, 215.1029, 227.0916, 249.1743, 263.1165, 271.1581, 277.1248, 279.0455, 288.1905, 300.1508, 314.1891, 318.1273, 340.1336, 378.1776, 380.1716, 395.1615, 413.2841, 445.1624, 453.1800, 461.1875, 463.1704, 477.2495, 479.1976, 481.1998, 497.2594, 514.2772, 516.3461, 558.3035, 562.2511, 576.2313, 578.2986, 580.2495, 582.3309, 594.2188, 610.2933, 627.3513, 656.2969, 679.2567, 709.1868, 712.3207, 729.2847, 739.2881, 741.3154, 757.1880, 757.3400, 790.4645, 792.3235, 812.3890, 828.4506, 831.4028, 838.2238, 840.1855, 856.3721, 875.4554, 893.4846, 903.5486, 915.4808, 949.5200, 955.3937, 955.5461, 957.4230, 1004.5699, 1038.3572, 1038.5460, 1052.5078, 1056.5205, 1057.6636, 1103.7076, 1151.6017, 1153.3240, 1202.7631, 1203.7820, 1321.7679, 1332.6829, 1333.6954, 1348.8071, 1365.8616, 1462.9999, 1463.8289, 1480.9071, 1582.0714, 1583.9395, 1640.0157, 1683.0854, 1813.1046, 1914.5978, 1915.7063)
1975.0325

Conclusion:

- No statistically significant protein was identified.

Sample: 3

Reflector positive ion mode MALDI-TOF-MS spectrum

(Select image and zoom in to see it correctly)

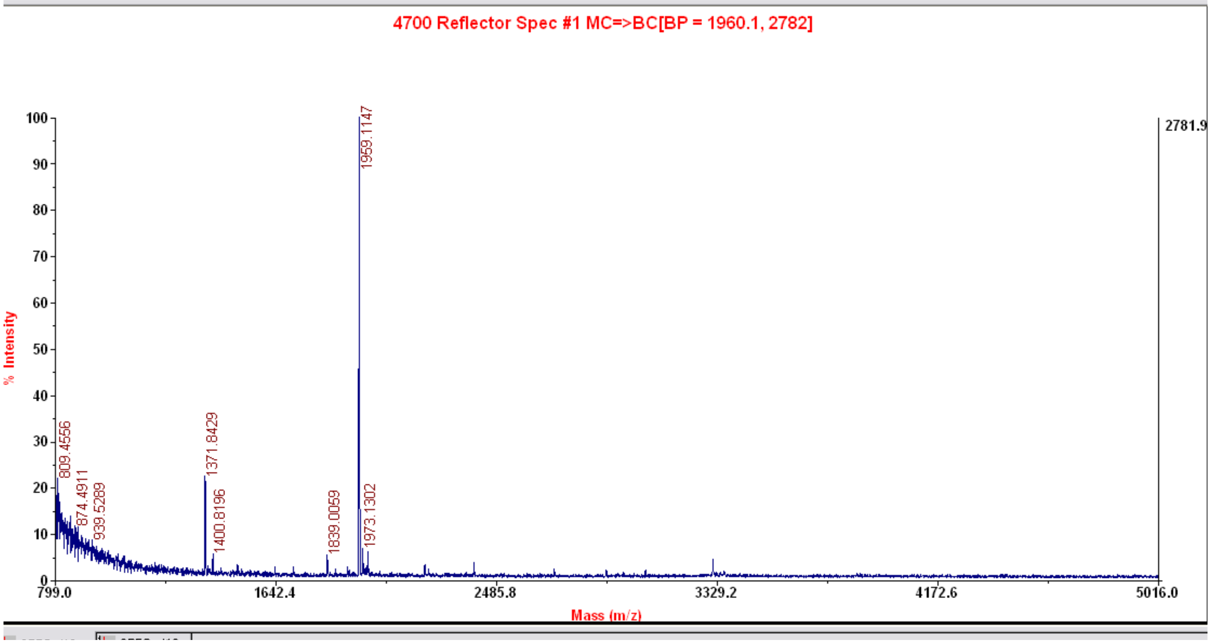

MS (m/z) y MS/MS (m/z) mass list obtained experimentally::

1371.7797, 1400.7695, 1495.8231, 1707.8016, 1838.9609, 1915.0375, 1957.0168
1959.0372 ions(175.1222, 199.1290, 215.1329, 227.0974, 271.1711, 277.1205, 288.1844, 314.1772, 340.1411, 378.1953, 380.1168, 463.1998, 477.2068, 479.1980, 481.1781, 497.1940, 497.3118, 514.2819, 578.3003, 580.2082, 594.2248, 610.3270, 627.2424, 627.4063, 693.2411, 709.3002, 729.2596, 729.3769, 757.1449, 757.3315, 790.3543, 790.5201, 791.3876, 792.5081, 828.4306, 840.2524, 856.3957, 857.2833, 903.5280, 955.4575, 1004.5115, 1004.6898, 1005.5563, 1038.5182, 1056.5693, 1103.6793, 1151.5457, 1202.7758, 1365.8810, 1463.8700, 1480.9338, 1581.9969, 1683.0656)
1973.0455, 1975.0388

Conclusion:

- No statistically significant protein was identified.

Sample: 4

Reflector positive ion mode MALDI-TOF-MS spectrum

(Select image and zoom in to see it correctly)

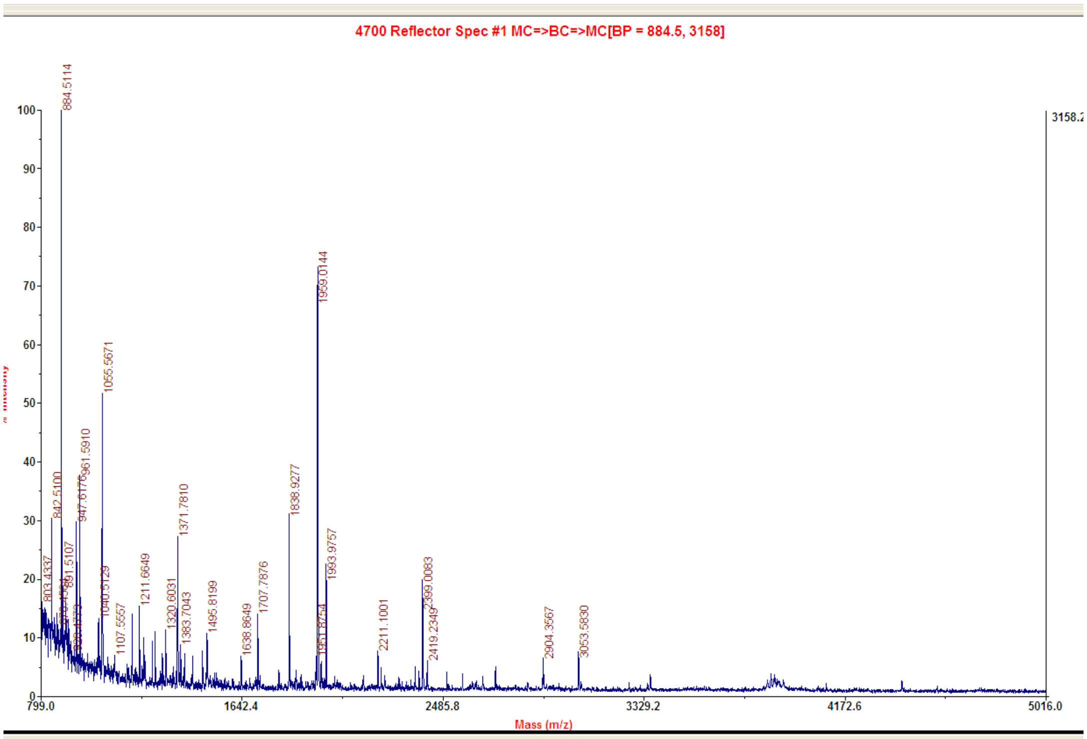

MS (m/z) y MS/MS (m/z) mass list obtained experimentally:

868.4529
884.4908 ions(171.1143, 175.1464, 186.1192, 228.1535, 229.1595, 256.1788, 257.1713, 284.1952, 286.1833, 295.1537, 303.2007, 341.2114, 363.2084, 365.1738, 367.2156, 384.2440, 389.2170, 390.2559, 395.2040, 412.2348, 414.2480, 431.2839, 454.3041, 590.3787, 611.4290, 646.3613, 651.2739, 697.4055, 712.4073, 714.4352, 716.3780, 768.4301, 811.6238, 812.5529, 813.5923, 820.4266, 822.4871, 824.4838, 826.4763, 840.4628)
886.4672, 887.4514, 891.4952, 900.4056, 961.5795, 1038.5323
1055.5581 ions(171.1105, 175.1347, 188.1249, 256.1781, 284.1779, 328.1733, 341.2166, 345.2118, 429.2826, 445.2270, 454.2617, 513.3289, 537.3370, 541.3013, 585.2961, 602.3479, 683.4312, 711.3697, 868.4629, 885.5336, 906.6250, 908.5636, 992.4900)
1056.5277, 1179.6061, 1211.6602, 1265.5901, 1278.7007, 1320.6130, 1371.7771, 1383.6863, 1475.7810, 1495.8187, 1707.7987, 1838.9482, 1951.8992
1959.0352 ions(175.1203, 215.1705, 249.0992, 271.1406, 277.1355, 288.1848, 290.1466, 378.0877, 445.1983, 463.2206, 497.2658, 514.2366, 514.3619, 527.2186, 558.2946, 578.1744, 580.3389, 582.3148, 594.2327, 610.2559, 627.3747, 679.3570, 729.2488, 730.2635, 732.2698, 741.1564, 742.2822, 757.1578, 757.4269, 790.4088, 791.5460, 829.2470, 856.3810, 857.4769, 903.5758, 957.6352, 1004.6078, 1038.5541, 1056.4384, 1057.6510, 1103.6395, 1153.7114, 1202.7650, 1365.8772, 1368.7775, 1480.9525, 1481.8163, 1683.0325, 1685.2117)
1993.9980, 2211.1257, 2399.0520, 2905.4224, 3053.6575

Conclusion:

- No statistically significant protein was identified.

Sample: 5

Reflector positive ion mode MALDI-TOF-MS spectrum

(Select image and zoom in to see it correctly)

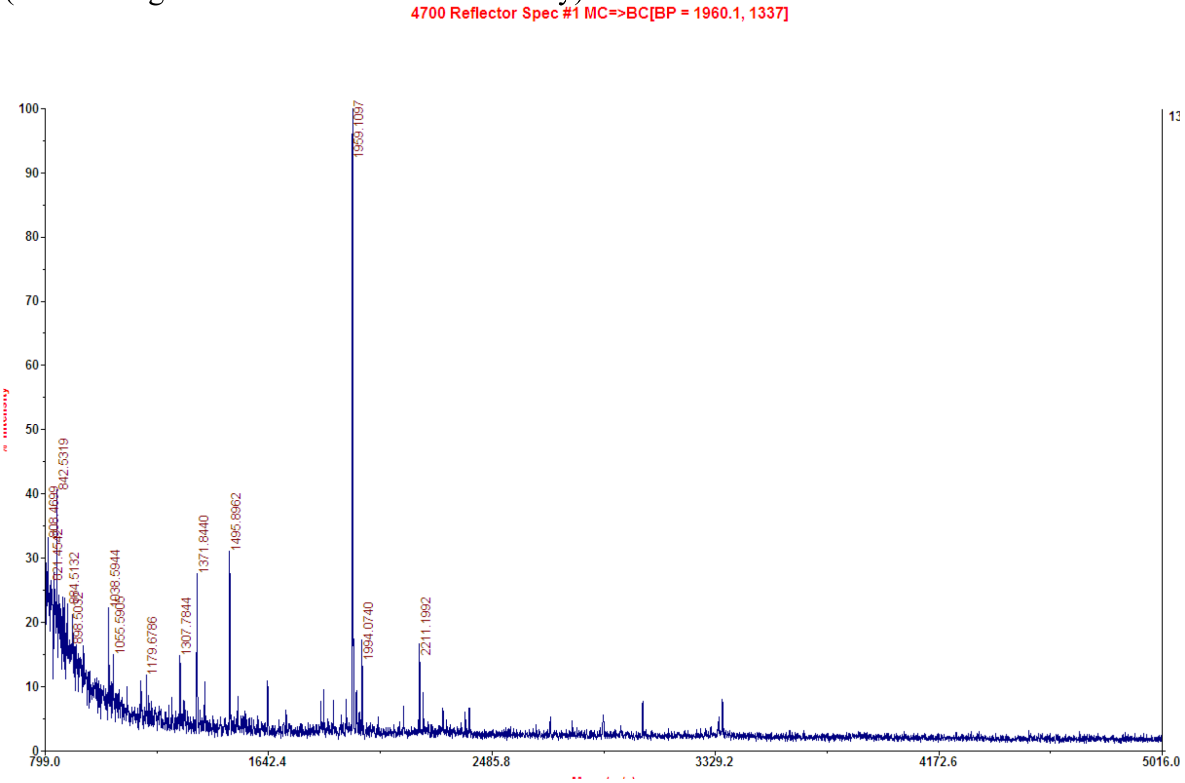

MS (m/z) y MS/MS (m/z) mass list obtained experimentally:

1372.7627
1495.8184 ions(175.1355, 213.1345, 229.1243, 244.1262, 263.1391, 274.2244, 278.1208, 329.1891, 357.1338, 362.2044, 377.1693, 387.2547, 407.1825, 438.2793, 506.2350, 516.3389, 597.3704, 605.2457, 615.3762, 617.2316, 619.3058, 639.3914, 669.3874, 728.5084, 729.4850, 751.4773, 768.2894, 768.4603, 769.4149, 771.2905, 788.4672, 857.4632, 882.5670, 1109.7258, 1134.6880)
1959.0205 ions(175.1243, 271.1523, 277.0856, 288.1880, 318.1306, 340.1360, 378.1677, 380.1582, 463.2169, 477.2075, 479.2289, 481.1703, 497.2910, 514.2830, 558.2936, 580.2449, 594.2537, 610.2335, 627.3727, 661.4327, 679.3425, 709.2445, 729.1758, 757.3518, 790.3795, 790.5095, 791.5193, 828.3796, 856.2413, 856.4561, 858.4203, 903.4185, 903.6609, 905.5907, 906.5305, 955.3461, 956.4108, 1004.6019, 1038.4539, 1046.4324, 1058.5426, 1126.4280, 1152.6526, 1154.4398, 1203.7170, 1365.0148, 1365.9116, 1463.8669, 1480.9143, 1683.2244, 1685.0482, 1788.9968)

Conclusion:

- No statistically significant protein was identified.

Sample: 6

Reflector positive ion mode MALDI-TOF-MS spectrum

(Select image and zoom in to see it correctly)

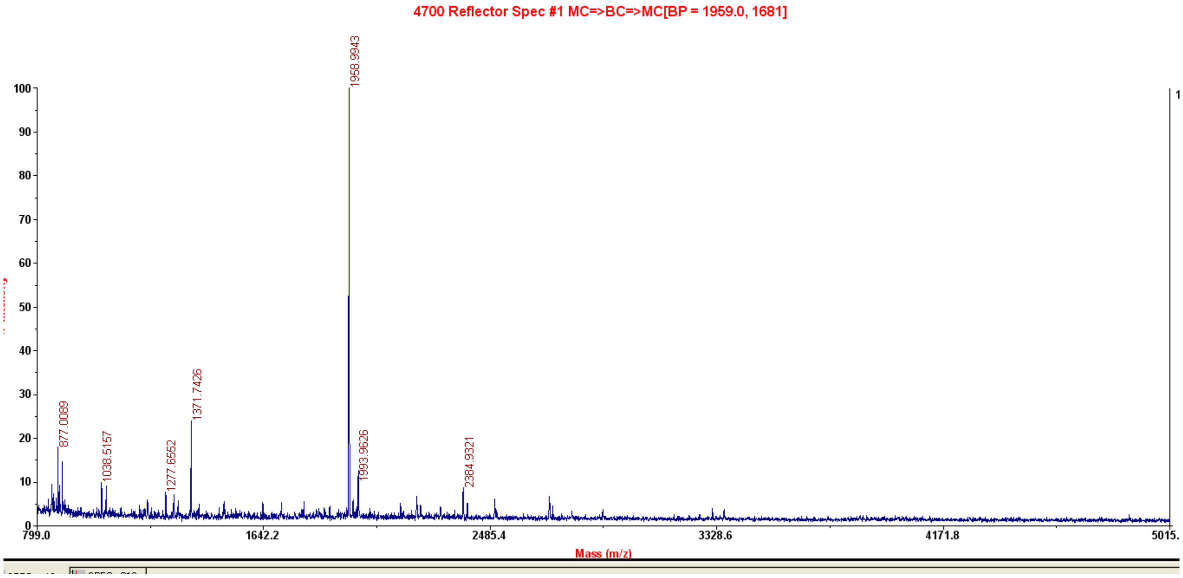

MS (m/z) y MS/MS (m/z) mass list obtained experimentally:

1038.5790, 1055.5940, 1209.6901, 1277.7274, 1307.7871, 1323.7034, 1371.8184, 1495.8723, 1638.9365, 1791.8146
1959.0906 ions(175.1341, 288.2188, 378.1609, 481.2440, 497.2274, 514.2897, 580.3113, 595.0085, 635.2825, 750.4213, 755.2444, 757.1755, 758.1273, 790.5102, 856.4193, 904.6568, 1074.1693, 1225.5076, 1366.8457, 1367.1294, 1369.9735, 1465.9008, 1683.0371, 1684.2501, 1783.0515, 1843.5929, 1936.1022)
1975.1095, 1994.0601, 2151.1838

Conclusion:

- No statistically significant protein was identified.

Sample: 7

Reflector positive ion mode MALDI-TOF-MS spectrum

(Select image and zoom in to see it correctly)

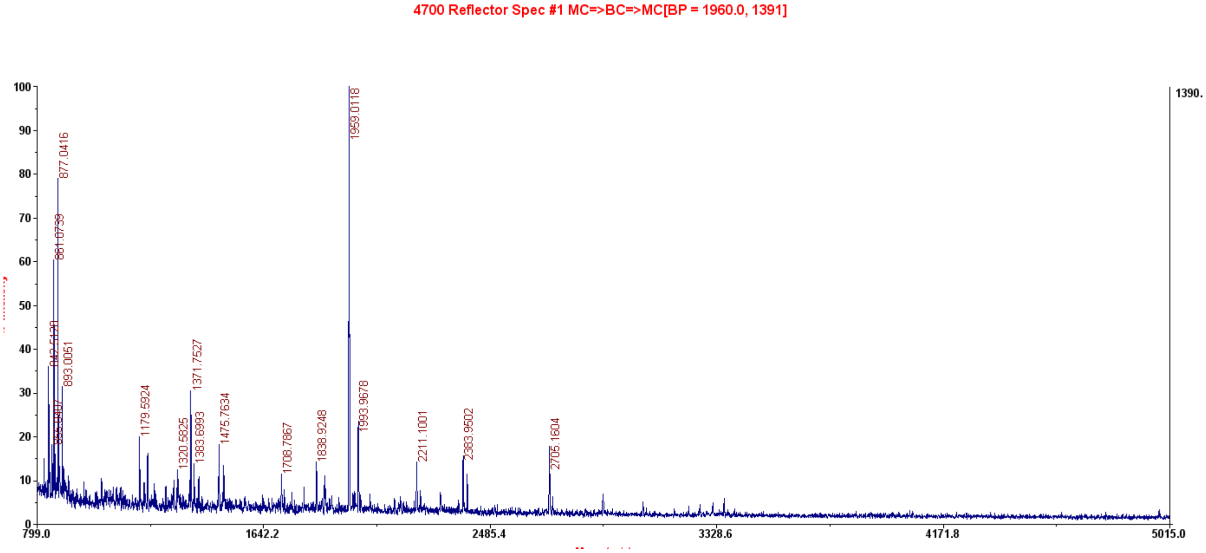

MS (m/z) y MS/MS (m/z) mass list obtained experimentally:

1209.6525
1320.5825
1371.7527 ions(175.1250, 486.8487, 560.4181, 646.3328, 692.4818, 702.3256, 787.3948, 827.3884, 926.8763, 938.7315, 1020.4038, 1028.9651, 1129.3838, 1181.9899, 1188.6985, 1220.7089, 1235.1007, 1247.8213, 1248.5891, 1260.7865, 1353.6530)
1383.6993, 1401.7311, 1475.7634, 1493.7509, 1708.7867, 1838.9248, 1868.9156
1959.0118 ions(175.1258, 227.1031, 288.2061, 497.2530, 514.3031, 601.1588, 627.5447, 650.1909, 710.3364, 757.3334, 856.4046, 1038.4635, 1059.7043, 1062.6381, 1365.7683, 1366.9445, 1747.4218)

Conclusion:

- No statistically significant protein was identified.
